# The influence of a low glycemic index dietary intervention on maternal dietary intake, glycemic index and gestational weight gain during pregnancy: a randomized controlled trial

**DOI:** 10.1186/1475-2891-12-140

**Published:** 2013-10-31

**Authors:** Ciara A McGowan, Jennifer M Walsh, Jacinta Byrne, Sinead Curran, Fionnuala M McAuliffe

**Affiliations:** 1 UCD Obstetrics and Gynaecology, School of Medicine and Medical Science, University College Dublin, National Materntiy Hospital, Dublin 2, Ireland; 2 National Materntiy Hospital, Dublin 2, Ireland; 3 Department of Nutrition and Dietetics, National Maternity Hospital, Dublin 2, Ireland

**Keywords:** Glycemic index, Pregnancy, Gestational weight gain

## Abstract

**Background:**

Maternal diet is known to impact pregnancy outcome. Following a low glycemic index (GI) diet during pregnancy has been shown to improve maternal glycemia and reduce infant birthweight and may be associated with a higher fibre intake. We assessed the impact of a low GI dietary intervention on maternal GI, nutritional intake and gestational weight gain (GWG) during pregnancy. Compliance and acceptability of the low GI diet was also examined.

**Method:**

Eight hundred women were randomised in early pregnancy to receive low GI and healthy eating dietary advice or to receive standard maternity care. The intervention group received dietary advice at a group education session before 22 weeks gestation. All women completed a 3 day food diary during each trimester of pregnancy. Two hundred and thirty five women from the intervention arm and 285 women from the control arm returned complete 3x3d FDs and were included in the present analysis.

**Results:**

Maternal GI was significantly reduced in the intervention group at trimester 2 and 3. The numbers of women within the lowest quartile of GI increased from 37% in trimester 1 to 52% in trimester 3 (P < 0.001) among the intervention group. The intervention group had significantly lower energy intake (P < 0.05), higher protein (% TE) (P < 0.01) and higher dietary fibre intake (P < 0.01) post intervention. Consumption of food groups with known high GI values were significantly reduced among the intervention group. Women in the intervention low GI group were less likely to exceed the Institute of Medicine’s GWG goals.

**Conclusion:**

A dietary intervention in early pregnancy had a positive influence on maternal GI, food and nutrient intakes and GWG. Following a low GI diet may be particularly beneficial for women at risk of exceeding the GWG goals for pregnancy.

**Trial registration:**

Current Controlled Trials Registration Number: ISRCTN54392969.

## Background

It is well documented that pregnancy is a critical period in a woman’s life where nutrition is of key importance for optimal pregnancy outcome [1,2]. There is also evidence that maternal weight and maternal gestational weight gain (GWG) exert a profound influence on infant birthweight. One of the main environmental factors regulating fetal growth is maternal substrate delivery to the placenta; thus, factors that modify maternal blood glucose levels can alter the rate of fetal growth [3,4]. Maternal diet, and particularly its carbohydrate (CHO) type and content, influences maternal blood glucose concentrations. However, different CHO foods produce different glycemic responses. The GI was conceived by Jenkins in 1981 as a method for assessing the glycemic responses of different CHO [5]. Data from clinical studies in non-diabetic healthy pregnant women have documented that consuming a low GI diet during pregnancy reduces peaks in postprandial glucose levels and normalises infant birth weight [6,7]. Pregnancy is a physiological condition where the GI may be of particular relevance as glucose is the primary fuel for fetal growth. A systematic review of GI and pregnancy in 2010 concluded that there remains insufficient evidence to recommend a low GI diet in normal pregnancies [8]. In pregnant women with GDM, following a low GI diet may reduce the need for insulin without adverse effects on pregnancy outcomes [9].

Little is known about the impact dietary interventions have on maternal dietary intake in pregnancy [10]. The primary aim of the present analysis was to examine the influence of a low GI dietary intervention on maternal GI and dietary intake. The secondary aims were to examine the effect on maternal GWG and to assess compliance and acceptability of the low GI diet in the intervention group.

## Methods

The ROLO (Randomized cOntrol trial of LOw glycemic index diet to prevent macrosomia in euglycemic women) study was carried out at the National Maternity Hospital (NMH), Dublin, Ireland. Dublin is Ireland’s capital city and has a population of 1.8 million people. This was a randomised controlled trial (RCT) of a low GI dietary intervention versus no dietary intervention in preventing recurrence of fetal macrosomia (http://www.controlled-trials.com; ISRCTN54392969). Ethical approval for the study was obtained from the National Maternity Hospital Ethics Committee in November 2006. Detailed methods and principal results of the ROLO study have been previously published [11,12]. In brief, the ROLO study used a presenting sample of secundigravid women who previously delivered a macrosomic infant weighing equal to or greater than 4000 g. They presented to the NMH for antenatal care between January 2007 and January 2011. Inclusion criteria included: ≥18 years of age, singleton pregnancy, between 10–18 weeks gestation, and adequate English to enable study participation. Women were excluded if they had previous or current gestational diabetes (GDM), taking medication for a known medical condition, or if they were pregnant with twins/triplets. Eligible women were identified by the hospital’s information officer and were contacted by telephone by the research midwife or dietitian. Interested parties were invited to the antenatal clinic at the NMH for a first antenatal hospital visit.

At this first antenatal visit, women’s medical history was taken along with routine blood tests and an ultrasound scan to confirm the pregnancy. All women were weighed by the research team in light clothing using a SECA weighing scales (SECA gmbh & co. Kg. Germany) to the nearest 0.1 kg and height was measured without shoes to the nearest 0.1 cm using a wall mounted stadiometer. 'Underweight’ was defined as having a body mass index (BMI) <18.5 kg/m^2^[13], 'normal weight’ as BMI 18.5 – 24.9 kg/m^2^; 'overweight’ as BMI 25 – 29.9 kg/m^2^; and 'obese’ as BMI ≥ 30 kg/m^2^[14]. Information on the study was given and written informed consent was obtained from all subjects to be included in the study. Subjects were then randomized into one of two groups (a 'low GI diet’ group and a 'control’ group). Randomization was achieved using computer-generated allocations in a ratio of 1:1 contained in sealed opaque envelopes. Randomization was carried out by an independent researcher at the NMH. The obstetricians and midwives who provided clinical care to participants were blinded to group assignment. It was not possible to blind study participants thus women in the control group understood that another group were receiving dietary advice. The control arm received standard care during their pregnancy which does not usually include formal dietary advice, although a booklet with standard information on healthy eating in pregnancy is available to all women at booking as part of their usual care. An independent data monitoring committee reviewed recruitment and safety of the ROLO study when 350 participants were recruited.

### Dietary intervention

Women randomized to the intervention group attended a dietary education session in groups of 2–6 with the research dietitian. The session lasted between 1–2 hrs and was carried out at least 2 weeks after the first antenatal visit to allow for completion of the first 3d FD. The diet was designed to meet nutritional recommendations for pregnant women [15]. Advice on the healthy eating guidelines for pregnancy following the food pyramid [15] and according to the Irish Nutrition and Dietetic Institute [16] were given. Women were not given specific information on their individual energy requirements or GWG goal for pregnancy, however, they were recommended not to 'eat for two’ and to consume an additional 200–300 calories/day in the last trimester of pregnancy.

The focus of the education session was the GI: its definition, concept and rationale for use in pregnancy. Women were given written information about the GI and a list of foods that were high and low in GI. They were encouraged to consume low GI CHO foods at their meals and to exchange high GI CHO foods for low GI alternatives. Women received a list of low GI recipes after the education session and additional low GI recipes were emailed to subjects during their pregnancy. The recipes were either taken from the internet or from the *'GI News’* newsletter produced from Professor Jennie Brand-Millers research team at the University of Sydney [17] (http://www.glycemicindex.com). Professor Jennie Brand-Miller’s book *'The New Glucose Revolution’* was recommended for any woman who was interested in learning more about GI [18]. There was no specific advice given regarding physical activity but when the issue was brought up the physical activity recommendations endorsed by the American Congress of Obstetricians and Gynaecologists (ACOG) and the Royal College of Obstetricians and Gynaecologists (RCOG) were advised [19,20].

To assess compliance and acceptability of the low GI diet, a questionnaire was given to patients in the intervention group at their 34 week antenatal visit. The compliance question was based on a 5-point Likert scale (no. 1 being 'I followed the recommended diet all of the time’ and no. 5 being 'I followed the recommended diet none of the time’). The acceptability questionnaire consisted of 6 questions exploring different aspects of how acceptable the diet was to follow and whether the changes made were accepted by the whole family. These questions were also based on a 5-point Likert scale (no. 1 being 'strongly disagree’ and no. 5 being 'strongly agree’) and were used in a previous study by Moses et al., 2006 [6].

### Dietary assessment method

All subjects completed a 3 day food diary (3d FD) during each trimester of pregnancy where the type and amount of all foods and beverages consumed were recorded over three consecutive days. Subjects were encouraged to include one weekend day during the recording period. Thirty five percent of women were recruited after the first trimester (15-18 weeks), therefore, their first trimester FD was actually completed in the early weeks of the second trimester. Subjects were instructed to quantify their food consumed using either the manufacturer’s weight on the food packaging or using household measures (e.g. tablespoons). If the portion size was not recorded clearly it was quantified by the research dietitian using the *Average Portion Sizes* according to the Food Standards Agency [21]. Dietary data were entered into WISP version 3.0 [22] (Tinuviel Software, Llanfechell, Anglesey, UK). The food composition tables in WISP are derived from the sixth edition of McCance and Widdowson’s *Food Composition Tables*[23]. The research dietitian was solely responsible for the collection, quantification, coding, and entry and checking of the food diaries. The FD’s were reviewed once per week to check for errors and to document the quality of the data. If there were any days missing in the FD this was also documented. The WISP system included an over range check for portion sizes, by generating a warning if a food weight was entered five times more than an average large portion. The research dietitian met with the patient or contacted the patient by telephone if any issues with the food diaries arose.

### Food groups

Seventeen food groups (FGs) existed in the WISP database at the time of data entry. In order to get a comprehensive depiction of the patient’s diets, we created 36 FGs. A food file was exported from the WISP database containing the nutritional information for all foods and beverages consumed over the recording period. Each food code in the food file was manually checked by the research dietitian to establish which FG was most appropriate (see Additional file 1: Table S1 for a list of the different foods within each food group). The FG’s were similar to those used in a previous Irish national dietary survey [24] but CHO containing FG’s such as 'breads’ were divided into smaller groups: 'white breads’, 'brown breads’ and 'wholemeal/wholegrain breads’ as the GI of these breads varies.

### Glycemic index values of foods

There was a total of 5395 food codes in the WISP GI databank of which 2838 (52.6%) had a 'null’ GI value at the beginning of the study. The existing GI values were based on measured GI values from the International GI Tables, 2002 [25]. We began updating our GI databank and our methodology was published in 2011 [26] at which time 56.9% of food codes had a GI value assigned using the most recently published GI values [27]. In 2011 using the same methodology, we updated the remaining food codes with researchers from the Institute of Food and Health at University College Dublin. Currently, only 10.6% of food codes do not have a GI value assigned, of which none were consumed in the current study. Additional file 1: Figure S1 illustrates the methods used in assigning and amended. GI values to the WISP database. The WISP database calculated each subject’s Glycemic Load (GL) based on the GI and CHO content of each food and beverage consumed using the formula: Daily GI/100 × amount of CHO (g). GI was then computed from each subject’s GL using the formula: GL/amount of CHO (g) × 100.

### Participant’s follow up

Pregnancy care was provided by the obstetricians and midwives at the NMH as per standard practice. Study participants met the research team at least two other occasions during their pregnancy; 28 and 34 weeks gestation. The research dietitian provided reinforcement of the low GI diet for the intervention subjects at these times and answered any questions. Subjects were weighed by the research team at both these visits and the hospital’s midwives at all other antenatal appointments. After delivery, pregnancy outcome information was obtained from the medical notes.

### Sample size

The primary outcome of the ROLO study was difference in infant birthweight between the intervention and control groups. Power analysis was performed by Professor Helen Colhoun. To detect a 0.25 SD difference in birth weight (equivalent to a 102 g difference in the birth weight between the groups) with 90% power, three hundred and sixty women were required in each group. Thus, the sample size was set at seven hundred and twenty. A sample of eight hundred women was considered sufficient to allow for patient drop out and possible loss to follow up. The secondary outcomes were difference in GWG and dietary intake between the groups and are presented in the present paper.

### Assessment of energy underreporting

Schofield equations were used to calculate basal metabolic rate (BMR) using the patient’s weight (kg) and age (years) [28]. Goldberg’s method was used to predict levels of energy (calorie/MJ) underreporting using the ratio of energy intake (EI) to estimated BMR [29] . A ratio of ≤ 1.2 may indicate underreporting and a ratio of < 0.9 is a sign of definite underreporting [30]. It was decided to run our analyses both with and without under-reporters. We divided our subjects into three reporting groups: 'definite under-reporters’ if their Goldberg’s ratio was ≤ 0.9; 'potential under-reporters’ if their Goldberg’s ratio was > 0.9 but ≤ 1.2 and 'normal reporters’ if their Goldberg’s ratio was >1.2 [31].

### Statistical analysis

All data analysis used the intention-to-treat principle where subjects were classified according to their randomly assigned group regardless of compliance or duration. Participants for whom dietary data were available are included in the present paper. All statistical analysis was carried out in PASW statistics version 18.0 (SPSS Inc., Chicago, IL, USA). Nutrients and foods were assessed for normality using the Kolmogorov-Smirnov test. Histograms were also created for each nutrient and food variable to graphically check for normality. All food variables were not normally distributed and were log transformed prior to analysis. Any nutrient that was not normally distributed and were also log transformed to improve normality. Independent samples *t*-tests were used to compare baseline maternal characteristics, nutrient and food intakes between groups. Differences in categorical variables were compared using the *χ*^2^ test. Results were considered statistically significant when P < 0.05.

## Results

### Subject characteristics

**Figure 1:**
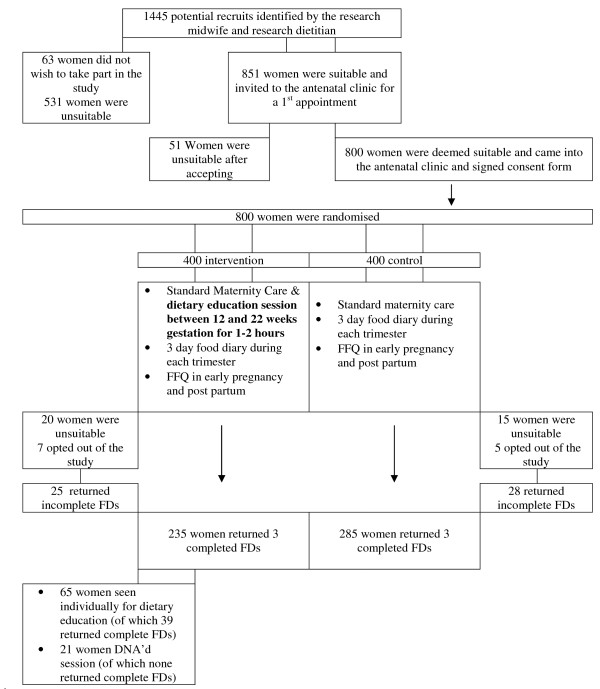
The flow of subjects in the ROLO study.

Figure 1 illustrates the flow of subjects through the study. During the study period 1,445 women who were in their second pregnancy presented to the NMH, having previously delivered an infant weighing equal to or greater than 4000 g. Of these, 531 were not contacted as they were met exclusion criteria from their previous pregnancy files. Of the remaining 914 who were contacted by telephone and informed of the study, 851 agreed to meet with a researcher. Of these, 51 were excluded and 800 were recruited and randomized. There was a similarly low drop-out rate in both groups, (7 vs. 5, in the intervention vs. control groups respectively). Women who opted out reported that their main reason was lack of time to attend hospital appointments. There were 19 early pregnancy (<14 weeks gestation) losses in the intervention arm and 14 in the control arm. There was one stillbirth in the intervention arm of an infant at 39 weeks gestation weighing 2.9 kg; post-mortem confirmed Trisomy 21. There was one mid trimester loss at 17 weeks in the control arm. These events were not deemed to be related to study participation. Five hundred and twenty women returned completed three × 3d FDs (70% of sample). A further 50 women returned incomplete data and were not included in the final analysis. There were no significant differences in maternal characteristics between those who returned full data and those who did not (data not presented). Table 1 reports the baseline maternal characteristics between the intervention and control groups, of which no significant differences were found.

**Table 1:** **Baseline characteristics at 1**^**st **^**antenatal visit between intervention and control groups**^**1**^

	**Intervention (n = 235)**	**Control (n = 285)**	**P**^**2**^
	**Mean ± SD**	**Mean ± SD**	
Age (years)	32.0 ± 3.8	31.7 ± 4.2	0.42
Gestation (wks)	12.8 ± 2.4	12.8 ± 2.3	0.65
Weight (kg)	72.5 ± 12.4	71.9 ± 12.4	0.64
Height (m)	1.66 ± 0.6	1.66 ± 0.6	0.37
BMI (kg/m^2^)	26.4 ± 4.4	26.3 ± 4.2	0.81
	**N (%)**	**N (%)**	
3^rd^ level Education^3^	132 (56.2)	150 (52.6)	0.46
Smoke	10 (4.3)	16 (5.6)	0.47
White Native Irish	209 (88.9)	246 (86.3)	0.24
Physically active^4^	59 (25.1)	62 (21.8)	0.37
Breastfeed 1^st^ child^5^	145 (61.7)	171 (60.0)	0.78

^1^The baseline/1^st^ antenatal visit was between 10 and 18 weeks gestation.

^2^P value assessed using independent samples *t*-test for continuous variables and χ^2^ test for categorical variables.

^3^Attainmet of a college/university bachelors degree or higher.

^4^Achieve 30 minutes of moderate intensity PA at least 5 days/week.

^5^Breastfeed their previous child for any length of time.

### Effect of the intervention

In total 21 women in the intervention group did not attend the dietary education session, of which none returned complete dietary data. Sixty nine women requested to be seen by the dietitian alone, of which 39 returned complete dietary data. Table 2 shows the change in mean dietary GI and the proportions of women within each quartile of GI during each stage of pregnancy. There was a significant but modest reduction in maternal GI among the intervention group. There were significantly more women in the intervention group within the lowest quartile of GI in trimester 2 and 3. At baseline there were no significant differences in energy or nutrient intake. Post dietary intervention, the intervention group reported significantly lower energy intake at both trimester 2 and 3 (7.6 MJ vs. 8.1 MJ, *P < 0.05*). They also had significantly higher intakes of dietary fibre (20.3 g vs. 18.8 g, *P < 0.01*), vitamin A (949.6 μg vs. 896.8 μg, *P < 0.05*) and magnesium (265.8 mg vs. 249.3 mg, *P < 0.001*) at trimester 3 (data not presented). After the dietary intervention, 10% fewer women in the intervention group consumed white bread and consumption of wholemeal and wholegrain breads increased from 67% in trimester 1 to 81% in trimester 2. Women in the control group were greater consumers of high energy beverages at trimester 2 (56% vs. 39%, *P < 0.001*) and refined breakfast cereals at both trimester 2 (57% vs. 42%, *P < 0.001)* and trimester 3 (60% vs. 42%, *P < 0.001*) (data not presented).

**Table 2:** Differences in mean ± SD GI and numbers of women within each quartile of GI between the intervention and control group from trimester 1 to trimester 3

	**Trimester 1**		**Trimester 2**		**Trimester 3**		**P**^**2**^
	**I**	**C**	**I**	**C**	**I**	**C**	
**GI**	57.3 ± 4	57.7 ± 4	56.1 ± 4	57.8 ± 4	56 ± 3.7	57.7 ± 3.9	<0.001
	P 0.25^1^		P <0.001^1^		P <0.001^1^		
				
	**N (%)**		**N (%)**		**N (%)**		
**Quartile GI**							
1*	37.4	32.2	50.2	30.5	51.5	31.2	
2	16.2	20.0	13.6	19.3	16.2	19.3	
3	23.4	19.3	18.3	22.1	17.9	23.9	
4	23.0	28.4	17.9	28.1	14.5	25.6	
	P 0.21^3^		P <0.001^3^		P <0.001^3^		

C, control group (n = 285); I, intervention group (n = 235).

*Quartile 1 (GI values 49.7 - 55.9); quartile 2 (GI values 56.0 -57.6); quartile 3 (GI values 57.7 – 59.9); quartile 4 (GI values 60.0 – 69.7).

^1^Differences in mean GI between groups across trimesters were compared using independent samples t-test.

^2^Differences in mean GI between trimester 1 and 2 and trimesters 1 and 3 within the intervention group assessed by ANOVA.

^3^Differences in numbers of women within each quartile of GI between groups were compared using the *χ*^2^ test.

Finally, Table 3 reports the numbers of women within each group who met, exceeded or were below the IOM’s GWG goals. Significantly more women in the control group exceeded the GWG goal (*P = 0.009*). Mean GWG was also significantly different between the groups (11.5 kg vs. 12.6 kg for the intervention and control group respectively, *P = 0.003*). There were no significant differences in the primary outcome (mean infant birthweight) between the two groups (4050 g vs. 4000 g in the intervention vs. control groups, p = 0.224). Approximately 50% of women in both groups went on to deliver a second macrosomic infant ≥4000 g [12].

**Table 3:** Maternal GWG between the intervention and control group

	**I**	**C**	***P***
	**Mean ± SD**	**Mean ± SD**	
Gestational weight gain (kg)	11.5 **±** 4.2	12.6 **±** 4.4	*0.003*
	**%**	**%**	
Meet IOM goal	39.3	34.9	0.31
Exceed IOM goal	33.2	44.7	*0.009*
Below IOM goal	27.5	20.4	0.06

C, control group (n = 285); I, intervention (n = 235).

P value assessed using the independent samples t-test for continuous data and using the *χ*^2^ test for categorical data.

### Effect of underreporting of energy

There were no significant differences in the prevalence of energy underreporting between the intervention and control groups. For the three trimesters combined, 43% of the intervention group and 38% of the control group had a Goldberg ratio ≤ 1.2 and were defined as 'potential underreporters’. Eleven percent and 9% of the intervention and control groups had a Goldberg ratio of ≤ 0.9 and were labelled as 'underreporters’. When 'underreporters’ (10% of the total sample) were excluded from the analysis, maternal GI in the intervention group was reduced from 57.1 in trimester 1 to 55.9 in trimester 2 and 3 (*P < 0.001*). Furthermore, when all 'potential underreporters’ (41% of the total sample) were removed from the dataset mean maternal GI in the intervention group was reduced from 57.1 in trimester 1 to 55.7 in trimester 2 (*P < 0.001*) and 55.4 in trimester 3 (*P < 0.001*). Statistically significant changes in maternal food and nutrient intakes were similar when 'underreporters’ or 'potential underreporters’ were removed from the analysis. When all 'potential underreporters’ were excluded mean GWG remained significantly different between the groups (12.1 kg vs. 13.2 kg in the intervention and control groups respectively, *P = 0.026*).

### Compliance and acceptability

Results from the compliance and acceptability questionnaires showed that 68% of women either agreed or strongly agreed that the diet was easy to follow. Sixty five percent of women agreed that they enjoyed making the changes to their diets, 72% of women reported that their family were happy with the changes they made to their diets, and 78% of women agreed/strongly agreed that they had enough energy while on the diet. Finally, over 80% of women reportedly enjoyed a wide variety of foods while following the diet.

## Discussion

A low GI dietary intervention in pregnancy significantly reduced maternal dietary GI and increased the numbers of women within the lowest quartile of GI. These changes were evident post intervention in the second trimester and they lasted into the third trimester.

A recent pilot trial by Rhodes et al. randomised 46 overweight or obese pregnant women to a low GL diet versus low fat diet. Maternal GI was significantly lower among the low GL group compared to the low fat group (GI: 52 vs. 58, p = 0.002) and dietary fibre intake was significantly higher among the low GL group (16.5 g vs. 13.4 g, p = 0.05). Reported energy intake among both groups was particularly low, approximately 1,600 kcal/day [32]. Under-reporting of energy intake is known to be higher among overweight and obese women and this may have explained the low energy intake reported.

Mean maternal GI at baseline was ~57 in both groups in the ROLO study. This is similar to the baseline GI in previous studies, including the European Prospective Investigation into Cancer and nutrition (EPIC) study carried out in 5 European countries [33]. Moses et al. reported a baseline GI of 58 in their sample of 72 pregnant women [6]. The mean GI of the American population is reported to be 57 [34]. After the low GI dietary intervention mean maternal dietary GI was significantly lower among the intervention group however, the actual difference was modest and its clinical significance is questionable (57.3 versus 56.1). Previous studies have reported larger decreases in maternal GI post intervention; however, these interventions employed more intensive dietary education sessions, often at numerous time points during pregnancy [6]. Reported energy intakes were lower in our intervention group and this has been found in other dietary intervention studies [6]. Energy intake was approximately 100 kcal lower among the low GI group in the trial by Moses and colleagues. Overall their energy intakes ranged from 1800 kcal to 1900 kcal, which is similar to the energy intakes reported in our study apriori exclusion of under reporters.

Women receiving the low GI dietary advice gained significantly less weight than the control group. On the contrary, Moses et al. reported greater but not statistically significant GWG in women following the low GI diet compared to a high GI diet [6]. Less GWG may be attributed to the lower reported energy intake in our intervention group. However, when underreporters were excluded from the analysis, GWG remained significantly higher among the control group. Our intervention did not include specific weight gain advice for women in the intervention group. In 2010, a meta-analysis of both randomized and non-randomized intervention study’s to reduce GWG found that dietary counselling and increasing physical activity combined with monitoring of weight gain during pregnancy appear to be successful in reducing GWG. The effects were particularly seen for preventing excess GWG beyond the IOM’s goals [35]. A more recent meta-analysis of RCTs to reduce GWG using lifestyle modifications also reported that positive changes in diet and physical activity were effective in reducing GWG. However, the authors called for stronger design of behaviour based GWG reduction interventions. Ten RCT’s were included in the meta-analysis but many underreported the content of their intervention and failed to report the effects on the subject’s dietary intake post intervention [10].

To our knowledge this is the first low GI dietary intervention study to be carried out in pregnant women who previously delivered a macrosomic infant. It is the largest RCT of low GI diet in pregnancy and is also the first study of its kind to report detailed food and nutrient intake data both pre and post dietary intervention. We presented detailed dietary data from three 3d FD’s which were completed at each trimester of pregnancy, both pre and post dietary intervention. Intakes of energy were significantly lower among the intervention group post intervention while intakes of dietary fibre and protein (% TE) were significantly higher among the intervention compared to the control group. We found that significantly more women in the intervention group consumed wholegrain breads and cereals, oily fish and yogurts while consumption of high energy beverages significantly reduced post intervention (data not presented). Rhodes et al. reported macronutrient and fibre intakes in their cohort but they did not record food or micronutrient intakes at baseline, prior to their intervention. Their dietary assessment method was two 24 hour recalls [32]. Earlier, Moses et al. also reported macronutrient and fibre intakes between their study groups but failed to mention micronutrient or food intakes. They used two 3d FD’s, two diet histories and two 24 hour recalls to assess maternal diet and GI [6,7]. Scholl et al. 2004, however, did report some micronutrient intakes in their observational study from 24 hour recalls. With the increasing prevalence of maternal obesity it has never been so important to monitor dietary intakes in pregnant women. Results from our study indicate that pregnant women appear to be receptive to information on healthy eating in pregnancy and may change their behaviour accordingly.

A possible limitation of the current study was that the low GI advice was only delivered once during pregnancy. All previous clinical trials of low GI diet and pregnancy delivered advice on a number of occasions [6,32]. Despite this, having a successful once-off dietary intervention would be more feasible to carry out on a larger scale. We also did not provide low GI CHO foods to our intervention group as was the case in previous studies [32,36]. It was also difficult to monitor patient compliance. Whilst keeping a food diary, the control group may have subconsciously or consciously changed their dietary behaviours. All participants taking part in this study including the control arm were aware that the study was examining the effects of diet during pregnancy. They were aware that their food records were being reviewed by a dietitian and this may have caused them to positively alter their diets.

## Conclusions

In conclusion, a low GI dietary intervention in early pregnancy significantly reduced maternal GI; increased dietary fibre intake; increased intake of wholegrain breads and cereals and reduced consumption of high energy beverages, white breads and refined cereals in a group of women who previously delivered a macrosomic infant. Those who received low GI dietary advice gained significantly less weight during pregnancy. Whilst the changes in maternal GI and weight gain may be model the present study along with future interventions of a low GI diet in pregnancy will help to establish who should receive low GI advice during pregnancy. The only systematic review of GI and pregnancy in 2010 called for larger-scale intervention studies of low GI diet in pregnancy. Results from our RCT would suggest that a low GI dietary intervention in early pregnancy may benefit women at risk of exceeding the GWG goals for pregnancy or those with a BMI in the overweight/obese category entering pregnancy. Pregnant women appear to be motivated to make positive changes to their diet during pregnancy.

## Consent

Written informed consent was obtained from the participant’s for the publication of the study’s results

## Abbreviations

GDM: Gestational diabetes mellitus; GI: Glycemic index; GL: Glycemic load; GWG: Gestational weight gain; IOM: Institute of medicine; ROLO: Randomised cOntrol trial of LOw glycemic index diet to prevent macrosomia in euglycemic women.

## Competing interests

The authors declare they have no competing interests.

## Authors’ contributions

FMM is the principal investigator of ROLO. CAM was responsible for patient recruitment, delivery of the dietary intervention, data entry and analysis and writing of the manuscript. JW was responsible for the running of the study and data analysis. JB was responsible for patient recruitment and running of the study. SC was involved in the design and delivery of the dietary intervention. All authors reviewed and revised the final version of the manuscript. All authors read and approved the final manuscript.

## Supplementary Material

Additional file 1 **Table S1.** The 36 food groups created and the foods within each food group. Figure S1: Outlining the methods used in assigning and amending GI values in the WISP database. Click here for file
