# Supplementary material for: The influence of a low glycemic index dietary intervention on maternal dietary intake, glycemic index and gestational weight gain during pregnancy: a randomized controlled trial
Source: Nutr J. 2013 Oct 31;12:140. doi: 10.1186/1475-2891-12-140 (PMC4176103; doi:10.1186/1475-2891-12-140)
Supplement: Additional file 1 — Table S1. The 36 food groups created and the foods within each food group. Figure S1: Outlining the methods used in assigning and amending GI values in the WISP database. [file 1475-2891-12-140-S1.doc]

| **Appendix Table 1. The 36 food groups created and the foods within each food group** | |
| --- | --- |
| **Food Group** | **Foods within each food group** |
| *White Bread and Rolls* | All breads and rolls made from white flour including French stick, Pitta, cream crackers, naan bread, ciabatta and chapatti |
| *Brown Bread and Rolls* | All breads and rolls made with brown flour including Irish soda bread |
| *Wholemeal/grain Breads* | All breads and rolls made from wholemeal/wholegrain flours including granary bread, rye bread and wholemeal crackers |
| *Pasta/Rice/Grains* | All pasta, rice, noodles and grains (couscous, quinoa, barley, buckwheat, bulgar wheat, cassava, tapioca) and flours (wheat/soya/rice/potato) |
| *Refined BF Cereals* | Cornflakes, Rice Krispies, Crunchy Nut Cornflakes, Special K, Frosties, Cocopops, Start, Sugar Puffs, Cereal Bars |
| *Wholegrain BF Cereals* | Branflakes, All-Bran, Wheat and Oat bran, Porridge, Oatflakes, Weetabix, Sultana Bran, Fruit’n Fibre, Cheerios, Ready Brek, Mueslis, Shredded Wheat |
| *Biscuits/Buns/Pastries/Cakes* | All biscuits (Digestives, Jaffa cakes), buns (Hot Cross buns, Currant buns), cakes (Sponge cake etc.) and pastries (Danish pastries, croissants etc.) |
| *Cream/Ice Cream/Deserts* | All types of cream, ice cream and deserts and puddings (apple pies, cheesecakes, custard, milkshakes, trifle, banoffee pie, crème caramel, mousse) |
| *Full Fat Milk* | All full fat cows/goats/sheeps/soya/rice milks |
| *Low Fat Milks* | All low fat and skimmed cows/goats/sheeps/soya milks |
| *Yogurts* | All yogurts including full and low fat varieties from cows/goats milk. |
| *Cheese* | All cheeses (Cheddar, Mozzarella, Brie, Camembert, Cream Cheese, Goats Cheese, Cheese spreads and processed cheeses) |
| *Sugars and Preserves* | All sugar (white/brown); jams, honey, molasses, treacle, salt and vinegar |
| *Confectionary* | All sweets, chocolate, chocolate bars and chocolate spreads |
| *Eggs and Egg Dishes* | All eggs (chickens/duck/quail) and egg dishes (omelette, quiche, etc) |
| *Butter/Full Fat Spreads* | All butter and spreads that contain > 40% fat |
| *Lower Fat Spreads* | All spreads that contain 40% fat or less |
| *Oils* | All oils (vegetable oils, olive oil, coconut oil, etc) |
| *Potatoes* | All boiled/baked/mashed potatoes including sweet potato, yam and plantain |
| *Chips* | All fried/roasted/chipped potatoes including potato wedges, potato cakes, potato fritters, potato waffles, hash browns, potato croquettes |
| *Beans/Lentils/Pulses* | All high protein vegetables including beans, bean products (tofu), lentils, pulses (peas, sweetcorn) |
| *Other Vegetables* | All root vegetables (carrots, parsnips) and green leafy vegetables. |
| *Fruit* | All kinds of fruit (fresh/canned/dried) |
| *Fruit Juice* | All kinds of fruit juice, freshly squeezed or bottled |
| *White fish and Shellfish* | White fish and shell fish (cod, haddock, plaice, whiting, canned tuna, crab, mussels, etc), Also includes products like fish pate and fish paste. |
| *Oily Fish* | Oily fish (salmon, trout, mackerel, eel, kipper, sardines, fresh tuna, swordfish, anchovies, herring etc) |
| *White Meat* | White meats (chicken, turkey, pork, pheasant, mutton, pigeon) |
| *Red Meat* | Red meats (beef, lamb, deer, duck, goose, rabbit) |
| *Other Meat/Meat Products* | Processed meats (bacon, ham, sausages, pudding); offal (liver and liver pate); meat products (nuggets, kebabs, burgers) and meat pies |
| *Savoury Snacks* | Savoury snacks such as potato crisps, popcorn, nuts, seeds, pretzels |
| *Sauces, Herbs, Spices* | Herbs (fresh and dried), spices, sauces and dressings (white sauces, pasta sauces, gravy, chutneys, relish) |
| *Soup* | Soups, broths and consommés |
| *Savouries* | Savoury dishes (Indian and Chinese dishes, Savoury Pancakes, Stews, Casseroles, Shepard’s Pie, etc.) |
| *Alcohol* | Includes all alcoholic beverages except non-alcoholic lager |
| *Low Energy Beverage* | Includes water, tea, coffee, sugar free cordials and diet fizzy drinks |
| *High Energy Beverages* | Includes non-diet fizzy drinks, fruit squashes/cordials, hot chocolate, malted drinks made on milk, etc |

| **Appendix Figure 1: Outlining the methods used in assigning and amending GI values in the WISP database** | | | | | | | |
| --- | --- | --- | --- | --- | --- | --- | --- |
|  | | | | | | | |
|  |  |  | 5395 food codes at beginning in the WISP nutrient databank  [2838 (52.6%) had a null GI value  [The remaining GI values were based on the 2002 International Tables] | |  |  |  |
|  |  |  |  |  |  |  |  |
| **Methodology**  Food codes were manually checked against the most up-to-date published tables (2008 International Tables) and assigned a new or amended GI value based on similar foods in the published tables | |  | First amending and assigning began in 2008 where 664 (12.3%) food codes had a GI value assigned or amended  [231(4.3%) food codes were assigned a GI value and 433 (8%) had a GI value amended] | |  | **Publication of methodology**: Levis SP, McGowan CA & McAuliffe FM (2011) Methodology for adding and amending GI values to a nutritional analysis package.  *Br J Nutr* **105**, 1117-1132 | |
|  |  |
|  |  |  |  |  |  |  |  |
| **Methodology**  1. Reviewed the current published GI tables, 2008, 2002 and 1995 and [www.glycemicindex.com](http://www.glycemicindex.com/)  2. Food codes were manually checked to see if they needed a GI value assigned or amended.  3.Values were assigned to foods that were similar to the foods in the published GI tables or if the GI value was based on UK studies  4.If the CHO content of the food was low (≤3g/100g) the GI assigned was zero | |  | Second amending and assigning completed in 2011 where  [2034 (37.7%) food codes were assigned a GI value and 119 (0.2%) food codes had a GI value amended] | |  | **Methodology cont.**  5. If the CHO content of the food was low (≤5g/100g) but there were significant amt of fat and/or protein the GI assigned was zero  6.For mixed recipes: first used the GI of the main CHO source; second, if there are a number of CHO sources in the meal the mean GI of these was used  7.Finally, if a food had not been tested for its GI but did contain CHO assign the value of 50 as per  Flood et al, 2006 | |
|  |  |
|  |  |  |  |  |  |  |  |
|  |  |  | Of the original 5395 food codes in the WISP databank only 573 food codes have a null GI value remaining | |  |  |  |
|  |  |  |  |  |  |  |  |
